# Performance evaluation of a novel screening tool for depressive episodes in COMISA: a comparative analysis with standard neuropsychometric assessments

**DOI:** 10.3389/frsle.2025.1648256

**Published:** 2025-09-11

**Authors:** Archie Defillo, Massimiliano Grassi, Silvia Daccò, Jennifer L. Martin, Daniela Caldirola, Giampaolo Perna

**Affiliations:** ^1^Clinical Research and Development Department, Medibio Limited, Savage, MN, United States; ^2^Department of Biomedical Sciences, Humanitas University, Milan, Italy; ^3^Department of Clinical Neurosciences, Villa San Benedetto Menni Hospital, Hermanas Hospitalarias, Albese con Cassano, Italy; ^4^Personalized Medicine Center for Anxiety and Panic Disorders, Humanitas San Pio X Hospital, Milan, Italy; ^5^VA Greater Los Angeles Healthcare System, University of California, Los Angeles, Los Angeles, CA, United States

**Keywords:** obstructive sleep apnea (OSA), insomnia, comorbid insomnia and sleep apnea (COMISA), current major depressive episode (cMDE), patient health questionnaire-9 (PHQ-9), mini-international neuropsychiatric interview, depression

## Abstract

**Introduction:**

Insomnia and obstructive sleep apnea (OSA) are each linked to elevated risks of depression. When comorbid (COMISA), these risks increase further, highlighting the need for effective depression screening. This study evaluated the screening accuracy of a novel software, MEB-001, for detecting current major depressive episode (cMDE) in individuals identified as with and without suspected COMISA (sCOMISA).

**Methods:**

We conducted a retrospective sub-analysis from a prospective multicenter study at U.S. sleep clinics, including 296 adults who underwent routine polysomnography (PSG). Electrocardiogram and electroencephalogram signals, along with items 1 and 2 of the self-report depression screener Patient Health Questionnaire, 9 items (PHQ-9), were used to generate MEB-001 screening output. The Mini International Neuropsychiatric Interview (MINI) served as the diagnostic reference for cMDE. Sensitivity, specificity, positive predictive value (PPV), and negative predictive value (NPV) were calculated for MEB-001 and PHQ-9 (cut-off ≥10), with subgroup comparisons conducted using Fisher's exact and McNemar's tests (*p* < 0.05).

**Results:**

MINI identified cMDE in 15.5% of participants (16.9% in sCOMISA; 14.2% in non-COMISA). Against the MINI, MEB-001 achieved 84.8% sensitivity, 72.0% specificity, 35.8% PPV, and 96.3% NPV in the full cohort; PHQ-9 ≥10 showed similar performance (89.1, 68.4, 34.2, and 97.2%, respectively). MEB-01's performance did not differ between sCOMISA and non-sCOMISA (all *p* ≥ 0.68), and no significant McNemar differences were found in the subgroups (*p* > 0.19).

**Discussion:**

MEB-001 demonstrated consistent cMDE screening performance comparable to the PHQ-9, supporting its potential utility in sleep clinic settings.

## 1 Introduction

Co-morbid insomnia and obstructive sleep apnea (COMISA) is a common and debilitating condition that leads to compounded difficulties in patients' sleep, daytime performance, and overall quality of life compared to those with either insomnia or obstructive sleep apnea (OSA) alone, making diagnosis and treatment decisions more complex for healthcare providers (Sweetman et al., 2019, 2017). Epidemiological findings reported that 39–58% of OSA patients report insomnia symptoms, and 29−67% of insomnia patients fulfill minimal criteria for OSA (Luyster et al., 2010). Between 2013 and 2018, several large cluster analyses of OSA samples revealed that 32% to 54% of patients experienced 'disturbed sleep,' primarily characterized by nocturnal insomnia (Ye et al., 2013; Gagnadoux et al., 2016). A recent systematic review and meta-analysis by Zhang and colleagues, which included 37 studies on insomnia and OSA co-morbidity, found that 35% of insomnia patients have an AHI ≥5 indicating the presence of mild OSA, 29% have an AHI ≥15 indicating the presence of a moderate condition, and 38% of OSA patients meet insomnia criteria (Zhang et al., 2019).

Both insomnia and OSA have been associated with psychiatric symptoms, including depression and anxiety (Luo et al., 2025). Studies reported that OSA patients report notably higher depressive symptoms compared to non-OSA cohorts, with prevalence estimates of between 17 and 48% (Harris et al., 2009). This is markedly elevated when compared to the global lifetime prevalence of depression, which ranges from 3 to 17% (Andrade et al., 2003).

Similarly, chronic insomnia frequently coexists with various psychiatric and physical disorders, notably depression. It often precedes and serves as a risk factor for the onset of depression and is recognized as both a risk factor and a symptom of the disorder. Consistent with this, studies have shown that treating insomnia, whether through pharmacotherapy or psychological interventions such as Cognitive Behavioral Therapy for Insomnia (CBT-I) (Koffel et al., 2018; Cheung et al., 2014; Qaseem et al., 2016; Araujo et al., 2017), can lead to significant reductions in depressive symptoms.

The burden of depressive symptoms associated with insomnia and OSA increases when these two conditions coexist. Smith et al. (2004) found that sleep clinic patients with comorbid insomnia reported moderate to severe depression, anxiety, and stress, unlike those with OSA alone, who showed non-clinical levels (Smith et al., 2004). Similarly, Lang et al. reported higher rates of depressive symptoms in individuals with COMISA (42.6%) compared to those with insomnia (21.6%) or OSA alone (8.4%), despite similar severity of sleep-related symptoms across groups (Lang et al., 2017).

More recently, data from the National Health and Nutrition Examination Survey indicated that individuals with probable COMISA were about three times more likely to report suicidal ideation and/or self-harm, with severe cases strongly associated with COMISA (Kalra et al., 2024).

A prospective study of 309 U.S. military personnel found that 32.7% had insomnia, 30.4% had OSA, and 36.9% had COMISA. Compared to those with OSA alone, individuals with insomnia or COMISA showed greater insomnia severity, poorer sleep quality, more disruptive nocturnal behaviors, and higher rates of PTSD, fatigue, anxiety, depression, and nightmare disorder (Mysliwiec et al., 2022).

Given the high comorbidity between COMISA and depression—and its significant impact on mental and physical wellbeing—depression in individuals with COMISA is a critical clinical concern. It amplifies the negative effects of sleep disturbances, contributing to poorer health outcomes. Effectively addressing depression in this population is essential to improving quality of life and mitigating related health risks. Emerging evidence suggests that individuals with COMISA face a greater risk of depression, anxiety, stress, and reduced quality of life compared to those with either insomnia or OSA alone (Sweetman et al., 2019, 2017; Smith et al., 2004; Lang et al., 2017; Krakow et al., 2001; Yang et al., 2011).

The U.S. Preventive Services Task Force (USPSTF) recommends depression screening for all adults who have not been previously diagnosed with a mental health disorder. This screening is typically conducted using the self-reported Patient Health Questionnaire, 9 items (PHQ-9) (Sheehan et al., 1998), which the USPSTF recognizes as the gold standard for the screening of current major depressive episode (cMDE) (Maurer et al., 2018; US Preventive Services Task Force, 2002; Mulvaney-Day et al., 2018).

Current depression screening tools rely on self-report measures, which, despite their general accuracy, are less effective in sleep clinic settings (Lequerica et al., 2022). For instance, Items 3 and 4 of the PHQ-9 may overestimate sleep issues, inflating scores and increasing false positives (Lequerica et al., 2022; Kroenke and Spitzer, 2002). Item 9, assessing suicidal ideation, raises legal and ethical concerns for clinicians. Additionally, mismatches between the PHQ-9′s two-week validity and delays in polysomnography (PSG) due to insurance can reduce screening accuracy (Ma et al., 2021).

While structured clinical interviews offer more reliable diagnoses, they require timely administration by trained professionals, adding to already strained clinical and financial resources. As such, an automated PSG-based depression screener could provide a more efficient, accurate, and timely alternative for identifying depression in sleep disorder populations.

To address the limitations of existing depression screening tools, this study evaluated the screening performance of a novel software, MEB-001, for detecting cMDE in individuals with and without suspected COMISA referred for sleep studies at multiple U.S. sleep clinics. MEB-001 utilizes ECG signals, EEG bands, sleep macrostructure, and responses to PHQ-9 Items 1 and 2, collected during routine inpatient PSG. Specifically, we examined whether combining physiological features with core depressive symptom reports, analyzed through machine learning algorithms, can effectively screen for cMDE at a level comparable to or better than the most widely used depression screening tool, the PHQ-9, when benchmarked against a structured clinical diagnostic reference.

We expect that MEB-001 will demonstrate high accuracy in screening for cMDE, performing comparably to, or exceeding, the PHQ-9 when validated against a structured clinical diagnostic interview.

## 2 Methods

A retrospective sub-analysis was conducted on data from a prior single-arm, prospective, multicenter trial known as “Sleep Signal Analysis for Current Major Depressive Episode (SAMDE),” registered with ClinicalTrials.gov (NCT05708222). This study aimed to evaluate the screening efficacy of MEB-001 for cMDE in individuals exhibiting and not exhibiting suspected comorbid insomnia and sleep apnea (COMISA) across a cohort of United States sleep clinics. This study received approval from the WGC Institutional Review Board (212 Carnegie Center, Suite 301, Princeton, NJ 08540 Tracking No. 20223322), and was conducted under the principles of the United States Food and Drug Administration (https://www.regulations.gov/docket/FDA-2011-D-0567).

Participants undergoing inpatient polysomnography (PSG) for suspected primary or secondary sleep disorders were enrolled based on predefined inclusion and exclusion criteria. Inclusion criteria were: age between 22 and 75 years; undergoing PSG for suspected sleep disorders—including, but not limited to, sleep-related breathing disorders, sleep-related movement disorders, circadian rhythm sleep-wake disorders (intrinsic and extrinsic), hypersomnia, parasomnia, and insomnia; ability and willingness to provide informed consent; and capacity to follow study procedures and instructions. Exclusion criteria included the presence of a pacemaker, history of heart transplantation, or undergoing a full-night C-PAP titration study.

The recruitment process commenced on June 6, 2023, and concluded on July 9, 2024, across multiple sites within the United States. Three sleep clinics were located in Texas, three in North Carolina, two in South Carolina, and one in Minnesota and Ohio, respectively.

Upon enrollment at the sleep clinic and immediately before their polysomnography (PSG) study, subjects completed a self-administered questionnaire collecting demographic and clinical information, including age, sex, medical history, comorbidities, and current medications. Subsequently, each participant completed the self-administered PHQ-9 via an electronic data capture (EDC) system on a tablet, from which the two essential items for depression screening were derived, serving as the comparator screening test for the algorithm development. The digital platform automatically generated a score, adhering to the PHQ-9 scoring guidelines established by Kroenke, Spitzer, and Williams in 2001. In addition, always before the PSG study, a fully structured interview was also administered to each patient, utilizing the Mini International Neuropsychiatric Interview (MINI). This interview was conducted by a certified, MINI-trained nurse practitioner via teleconference and aimed to diagnose the cMDE, which served as the reference standard for evaluating the screening performance of MEB-001. The nurse practitioners who administered the MINI were blind to each participant's PHQ-9 score and the MEB-001 output.

### 2.1 Mini international neuropsychiatric interview

The MINI Adult Version (Sheehan et al., 1998) is a structured diagnostic interview designed to assess major psychiatric disorders based on the DSM-5 (American Psychiatric Association, 2022) and ICD-10 (International Classification of Diseases, 2024; World Health Organization, 1993).

Widely used in mental health settings worldwide, it provides a brief yet comprehensive tool for efficiently identifying conditions such as mood, anxiety, psychotic, and substance use disorders. The interview consists of standardized yes/no questions, allowing for rapid administration in clinical and research contexts. Each disorder is evaluated through specific modules, with questions aligned with DSM-5 criteria for accurate diagnosis.

Validation and reliability studies of the MINI demonstrated a sensitivity of 0.96, a specificity of 0.88, a positive predictive value of 0.87, a negative predictive value of 0.97, an inter-rater Kappa of 1.00, and a test/retest Kappa of 0.87 in the diagnosis of major depressive disorder (MDD) (Sheehan et al., 1998) when compared to clinician-administered semi-structured interviews, specifically the Structured Clinical Interview (SCID) (Spitzer et al., 1992).

Comparisons between the Mini-International Neuropsychiatric Interview (MINI) and unstructured interviews conducted by mood disorder specialists have demonstrated a sensitivity of 0.87, a specificity of 0.63, a positive predictive value of 0.84, a negative predictive value of 0.68, and a Cohen's kappa of 0.51 (Rodrigues et al., 2024). Furthermore, the MINI has exhibited robust inter-rater reliability, even when administered by individuals without specialized mental health expertise (Sheehan et al., 1998; Rodrigues et al., 2024; de Azevedo Marques and Zuardi, 2008).

### 2.2 Patient health questionnaire (PHQ-9)

The PHQ-9 (Spitzer et al., 1999; Kroenke et al., 2001) is a validated 9-item self-report instrument widely utilized in the evaluation of depression across major sociodemographic segments within the U.S., with no significant variations in performance observed among these groups.

The nine items correspond to the nine DSM-5 criteria for a major depressive episode, as defined by the American Psychiatric Association (2022). Response options for each item are scored on a scale from “not at all” (0) to “nearly every day” (Luyster et al., 2010), quantifying the frequency with which each symptom has affected the respondent within the preceding 2-week period. The score derived from the nine items ranges from 0 to 27. Scores of 5, 10, 15, and 20 are indicative of mild, moderate, moderately severe, and severe depression symptoms, respectively. A score of 10 or higher is considered a threshold, signifying a potentially clinically significant condition, specifically a possible cMDE.

A recent and extensive Individual Participant Data Meta-analysis (Negeri et al., 2021), encompassing approximately 44,500 participants, evaluated the accuracy of the PHQ-9 in identifying cMDE by comparison with established reference standards. Specifically, in comparison to semi-structured psychiatric diagnostic interviews, sensitivity and specificity for a cutoff of ≥ 10 (95% CI) were determined to be 0.88 (0.82–0.92) and 0.86 (0.82–0.88), respectively. In the context of fully structured interviews, sensitivity ranged from 0.67 (0.57–0.76) to 0.75 (0.66–0.82), while specificity ranged from 0.86 (0.80–0.90) to 0.88 (0.84–0.91). In general, the PHQ-9 demonstrated acceptable accuracy in detecting depressive episodes. Concurrently, a score of ≥ 10 has been associated with an increased risk of major depression by a factor exceeding 2.6 (Kroenke et al., 2001).

Based on the substantial body of scientific evidence regarding the PHQ-9, the application of this cut-off threshold (≥ 10) is recommended as the most reliable approach for screening purposes in clinical practice and clinical trials (He et al., 2020).

### 2.3 OSA, suspected insomnia, and suspected COMISA determination

OSA was diagnosed based on the final clinical assessment made by a sleep specialist following interpretation of polysomnography (PSG) results. Insomnia symptoms were evaluated using a self-administered clinical questionnaire completed prior to the sleep study. The questionnaire included four items addressing sleep difficulties: trouble initiating or falling asleep, frequent nocturnal awakenings, early morning awakenings, and difficulty returning to sleep after nighttime awakenings. Each item was rated on a four-point Likert scale (“Never,” “Sometimes,” “Often,” “Always”). Participants reporting at least one of these symptoms as occurring “Often” or “Always” were classified as having suspected insomnia. Suspected COMISA (co-morbid insomnia and sleep apnea) was identified when both OSA and suspected insomnia criteria were met. Because insomnia was assessed via self-report rather than clinical evaluation, the COMISA classification should be considered provisional.

The Insomnia Severity Index (ISI), commonly used to identify insomnia in population-based samples and evaluate treatment response, and the Epworth Sleepiness Scale (ESS), used to assess daytime sleepiness, were not administered. As no formal clinical diagnosis of insomnia was performed, both insomnia and COMISA were considered suspected rather than confirmed diagnoses.

### 2.4 European data format (EDF) files

The physiological data recorded during PSG were extracted as EDF files. These files comprised full-night digital PSG recordings, conducted in accordance with the American Academy of Sleep Medicine (AASM) guidelines (American Academy of Sleep Medicine, 2023) and acquired utilizing a variety of PSG systems, including the SomnoStar 10.2 Sleep Scoring System (Vyaire Medical, Inc.), Philips Respironics Sleepware (G3), Natus Neurology Sandman Elite (10.1), and Compumedics Profusion PSG4 (V4.5).

Of the multiple physiological channels recorded during PSG, six electroencephalography (EEG) montages (F4A1, C4A1, O2A1, F3A2, C3A2, and O1A2; minimum sampling rate: 200 Hz) and the lead-II electrocardiography (ECG) channel (minimum sampling rate: 200 Hz) were employed as input for the MEB-001 software. All EDF and associated files underwent de-identification through the removal of personally identifiable information and were assigned a sequential identification code prior to collection.

### 2.5 MEB-001 software

**Figure 1:**
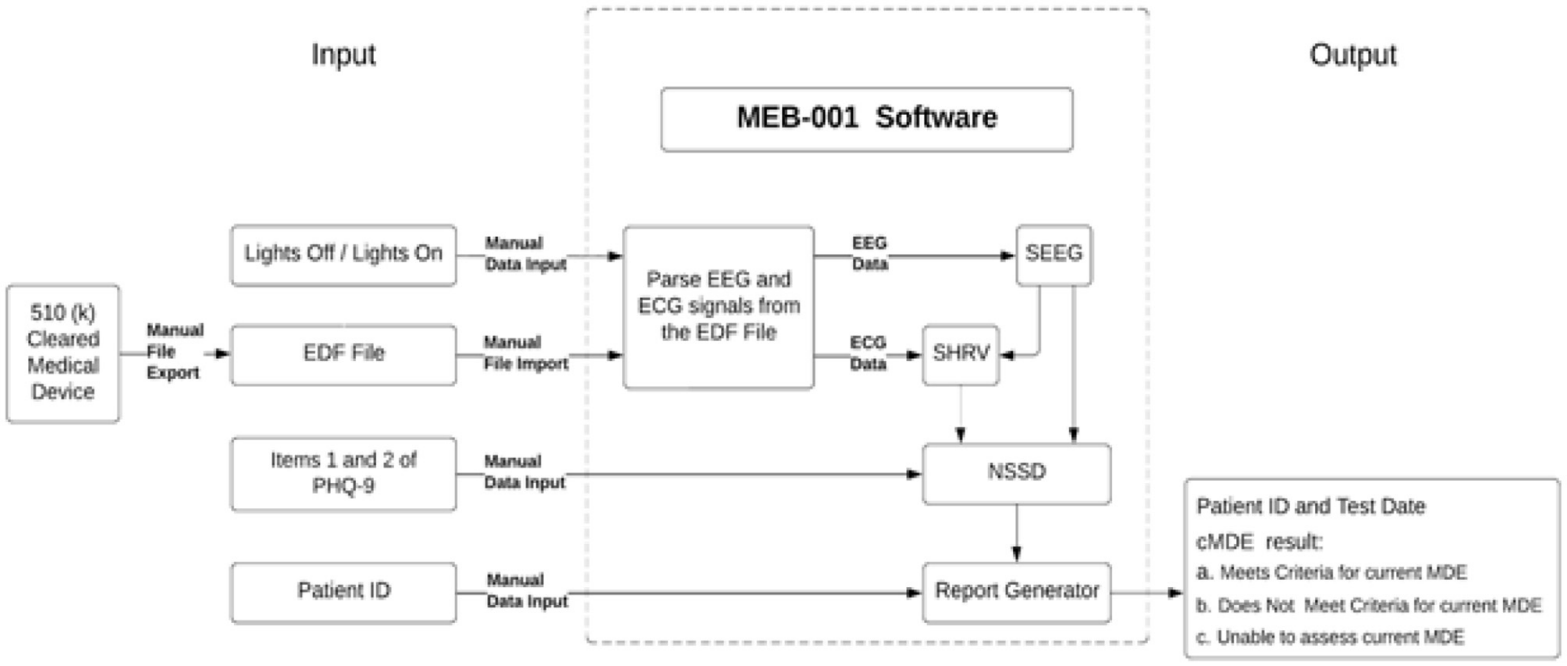
MEB-001 software overview. The MEB-001 diagram illustrates the software inputs and processing, which integrate EEG and ECG signals from PSG with Items 1 and 2 from the PHQ-9 to screen for cMDE in sleep centers. The three processing modules, SEEG, SHRV, and NSSD, are responsible for sleep staging based on EEG signals, calculating heart rate and heart rate variability throughout sleep stages, and determining the final cMDE, respectively.

This study presents a novel technology for the objective screening of active depressive symptoms in sleep centers, utilizing EEG and electrocardiogram (ECG) signals derived from PSG, in conjunction with two key components of the PHQ-9, specifically items 1 and 2 (Figure 1).

MEB-001, a software application currently in development, is designed to screen for cMDE in adult patients aged 22 to 75 years who are undergoing PSG for suspected sleep apnea. The software analyzes physiological data recorded during PSG, specifically six EEG derivations—three primary (F4A1, C4A1, O2A1) and three backup (F3A2, C3A2, O1A2)—as per the guidelines established by the AASM (American Academy of Sleep Medicine, 2023), and the lead-II ECG derivation. These signals are extracted from EDF files containing the complete physiological recordings from the PSG. A minimum sampling rate of 200 Hz is required for both EEG and ECG derivations. Furthermore, MEB-001 incorporates patient responses to PHQ-9 items 1 and 2, which are collected prior to the PSG. The software also necessitates the input of “lights-off” and “lights-on” times to delineate the initiation and termination of the sleep study.

The software commences its analysis with an automated sleep staging derived from six EEG channels. This module utilizes an automated sleep staging algorithm, which, as demonstrated in a prior investigation, exhibited substantial concordance with manual sleep staging conducted by independent sleep technologists (Grassi et al., 2023). The EEG signals undergo processing via a sequence of artificial intelligence, specifically machine learning and deep learning, algorithms that categorize each 30-s epoch into one of five sleep stages: wakefulness, N1, N2, N3, and REM. Concurrently, spectral analysis of the EEG data is executed to generate sleep macrostructure indices that depict sleep patterns observed throughout the polysomnography. Subsequent to sleep staging, the ensuing processing module incorporates ECG data to ascertain heart rate (HR) and heart rate variability (HRV) across discrete sleep stages. A range of HRV indices, encompassing time-domain, frequency-domain, and non-linear domain metrics, are computed for each respective stage.

In the final processing phase, select EEG and ECG-derived variables, previously obtained, are integrated with scores from PHQ-9 items 1 and 2. An ensemble of machine learning algorithms analyzes these inputs, subsequently generating a binary classification of cMDE status. In the event that EEG or ECG signals are significantly corrupted or deemed unusable, MEB-001 will produce an “Unable to calculate cMDE” output.

The machine learning algorithm employed in this module was trained using data acquired during Phase 1 of the Sleep Analysis Major of Depression Episode (SAMDE) study. Notably, the datasets utilized for the development and training of the machine learning algorithms, which constitute the various modules of MEB-001, were distinct from the dataset employed for testing in this particular study. The algorithms were finalized prior to the commencement of the study analysis and remained unaltered following exposure to the study data. Certain sleep clinics engaged in this study, specifically those located in Minnesota, Ohio, North Carolina, and South Carolina, also contributed to data acquisition during the software's developmental phase, whereas the clinics situated in Texas did not participate in such data collection.

### 2.6 Statistical analysis

The screening accuracy of the MEB-001 software and the PHQ-9, relative to the MINI, was evaluated using sensitivity, specificity, positive predictive value (PPV), and negative predictive value (NPV). Sensitivity was defined as the proportion of true positives (TP) among all individuals with the condition, calculated as TP/(TP + FN). Specificity referred to the proportion of true negatives (TN) among individuals without the condition, calculated as TN/(TN + FP). The PPV was the proportion of true positives among all test-positive cases, calculated as TP/(TP + FP), while the NPV represented the proportion of true negatives among all test-negative cases, calculated as TN/(TN + FN). In these formulas, FN refers to false negatives and FP to false positives. Exact 95% confidence intervals (CIs) were computed using the Clopper–Pearson (exact binomial) method.

Analyses were conducted on the full sample and stratified by OSA, suspected COMISA, and suspected insomnia diagnoses groups. Fisher's exact test was employed to compare sensitivity, specificity, PPV, and NPV between subgroups. For each metric, 2 × 2 contingency tables were constructed separately for individuals with and without suspected COMISA, based on classifications from the screening tools relative to the MINI. Sensitivity comparisons assessed differences in the proportion of true positives among MINI-positive cases (TP + FN); specificity comparisons examined the proportion of true negatives among MINI-negative cases (TN + FP); PPV comparisons evaluated the proportion of true positives among test-positive cases (TP + FP); and NPV comparisons assessed the proportion of true negatives among test-negative cases (TN + FN).

Additionally, McNemar's test was used to compare the paired screening performance of MEB-001 and PHQ-9. The analysis was stratified by cMDE diagnosis according to the MINI, and discordant classifications between the two tools were examined among true positive and true negative cases. This analysis was also conducted separately within the two COMISA subgroups.

All statistical tests were two-sided, with a significance threshold set at p < 0.05. Analyses were performed using Python (version 3.12.1) (Python Software Foundation, 2024).

## 3 Results

A total of 395 participants satisfied the study's inclusion and exclusion criteria and provided informed consent. Of these, 29 individuals were excluded based on clinical considerations. Conversely, 40 individuals were excluded as a result of PSG-related technical deficiencies, including but not limited to suboptimal signal quality or the recording of less than 6 h of data. Consequently, a final sample of 326 subjects was deemed usable for the study. These 326 subjects were recruited from multiple sleep clinics throughout the United States, specifically: three clinics in Texas (*n* = 62, 60, 31), three in North Carolina (*n* = 28, 3, 2), two in South Carolina (*n* = 95, 32), one in Minnesota (*n* = 10), and one in Ohio (*n* = 3).

**Figure 2:**
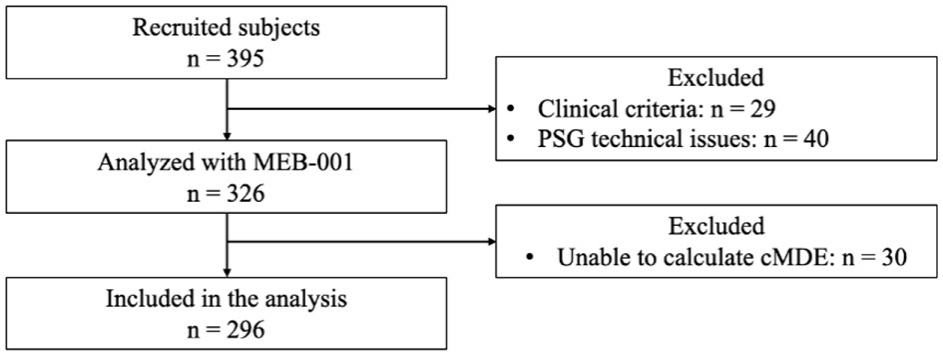
Study flowchart.

Thirty cases (9.23% of the processed data) received an “Unable to assess current cMDE” determination from MEB-001 due to significant signal anomalies. Specifically, issues were identified in EEG and sleep staging for 8 cases (2.46%) and in ECG and HR/HRV data for 22 cases (6.77%). As a result, these 30 cases were excluded from further analysis. The remaining 296 cases were used to evaluate the performance of MEB-001 (Figure 2). A detailed summary of their characteristics, including cMDE prevalence based on the MINI and PHQ-9 assessments, is provided in Table 1.

**Table 1:** Descriptive statistics.

**Demographic & Sleep Attributes**		**Total sample (*****n*** = **296)**		**Suspected COMISA (*****n*** = **148)**		**Not suspected COMISA (*****n*** = **148)**		**Unable to assess cMDE (*****n*** = **30)**	
		**Mean**	**S.D**.	**Mean**	**S.D**.	**Mean**	**S.D**.	**Mean**	**S.D**.
Age		41.11	12.50	42.90	12.85	39.32	11.91	44.47	15.68
BMI		30.23	7.75	30.98	8.38	29.47	7.01	28.78	8.35
Height in inches		67.32	4.23	67.15	4.25	67.49	4.22	66.27	3.65
Weight in lbs.		195.08	51.24	199.13	55.30	191.06	46.70	180.00	53.45
Total Sleep Time (minutes)		336.28	62.90	332.77	58.22	339.80	67.27	292.76	82.40
Apnea-Hypopnea Index		17.61	19.54	22.57	20.79	12.65	16.86	23.00	22.46
Sleep Efficiency		80.79	12.87	80.06	11.66	81.52	13.97	69.35	17.10
SpO2 < 88% (percent)		1.92	5.62	2.53	6.28	1.31	4.82	2.26	5.50
Respiratory Arousal Index		12.36	14.35	16.16	15.90	8.57	11.47	17.00	16.77
Periodic Leg Movement Arousal Index		1.01	2.98	0.85	2.95	1.17	3.01	0.21	0.47
Spontaneous Arousal Index		5.06	5.27	5.09	5.67	5.03	4.86	5.10	7.34
Total arousal index		19.31	15.07	22.90	16.31	15.73	12.80	23.07	17.45
PHQ-9 total score		8.76	5.74	9.68	6.01	7.84	5.33	8.63	5.85
**Demographic Attributes**		**N**	**%**	**N**	**%**	**N**	**%**	**N**	**%**
Gender	Female	154	52.03%	81	54.73%	87	58.78%	19	63.33%
	Male	140	47.30%	67	45.27%	59	39.86%	11	36.67%
	Other	2	0.68%	0	0.00%	2	1.35%	0	0.00%
Education	Some High School	2	0.68%	0	0.00%	2	1.35%	2	6.67%
	High School Diploma	59	19.93%	25	16.89%	34	22.97%	5	16.67%
	Associate degree	41	13.85%	19	12.84%	22	14.86%	4	13.33%
	Some Undergraduate School	29	9.80%	10	6.76%	19	12.84%	4	13.33%
	Bachelor's Degree	77	26.01%	47	31.76%	30	20.27%	8	26.67%
	Some Graduate School	20	6.76%	11	7.43%	9	6.08%	1	3.33%
	Graduate Degree	61	20.61%	32	21.62%	29	19.59%	5	16.67%
	Other	7	2.36%	4	2.70%	3	2.03%	1	3.33%
**Medical & Sleep Attributes**		**Total sample (*****n*** = **296)**		**Suspected COMISA (*****n*** = **148)**		**Not Suspected COMISA (*****n*** = **148)**		**Unable to assess cMDE (*****n*** = **30)**	
		**N**	**%**	**N**	**%**	**N**	**%**	**N**	**%**
Obstructive sleep Apnea	Mild obstructive sleep apnea (AHI between 5 and 15)	99	33.45%	70	47.30%	29	19.59%	10	33.33%
	Moderate obstructive sleep apnea (AHI Between 15 and 30)	71	23.99%	37	25.00%	34	22.97%	6	20.00%
	Severe obstructive sleep apnea (AHI >30)	54	18.24%	41	27.70%	13	8.78%	8	26.67%
	OSA Diagnosis	224	75.68%	148	100%	76	51.35%	24	80.00%
Central sleep apnea	Central Sleep Apnea	3	1.01%	1	0.68%	2	1.35%	0	0.00%
Suspected insomnia	Suspected Insomnia	203	68.58%	148	100%	55	37.16%	20	66.67%
Medical history	Diabetes - Type I	2	0.68%	0	0.00%	2	1.35%	0	0.00%
	Diabetes - Type II	9	3.04%	7	4.73%	2	1.35%	0	0.00%
	Insomnia	1	0.34%	1	0.68%	0	0.00%	0	0.00%
	Hypersomnia	0	0.00%	0	0.00%	0	0.00%	0	0.00%
	Hypertension	104	35.14%	52	35.14%	52	35.14%	18	60.00%
Body mass index	Underweight and healthy weight (BMI of 24.9 or less)	68	22.97%	33	22.30%	35	23.65%	8	26.67%
	Overweight (BMI of 25-29.9)	94	31.76%	47	31.76%	47	31.76%	6	20.00%
	Obesity (BMI of 30–39.9)	106	35.81%	51	34.46%	55	37.16%	4	13.33%
	Severe Obesity (BMI of 40 or more)	28	9.46%	17	11.49%	11	7.43%	12	40.00%
Cardiac issues	No issue	279	94.26%	138	93.24%	141	95.27%	27	90.00%
	PVCs	11	3.72%	6	4.05%	5	3.38%	2	6.67%
	A-Fib	3	1.01%	3	2.03%	1	0.68%	1	3.33%
	PACs	3	1.01%	2	1.35%	1	0.68%	0	0.00%
	CAD	1	0.34%	0	0.00%	0	0.00%	0	0.00%
	Bradycardia	0	0.00%	0	0.00%	0	0.00%	0	0.00%
	Tachycardia	0	0.00%	0	0.00%	0	0.00%	0	0.00%
Positive cMDE determination	MEB-001	109	36.82%	51	34.46%	58	39.19%	-	-
	PHQ-9 (cut-off ≥ 10)	120	40.54%	54	36.49%	66	44.59%	9	30.00%
	MINI	46	15.54%	25	16.89%	21	14.19%	4	13.33%

A-Fib, atrial fibrillation; AHI, apnea-hypopnea index; BMI, body mass index; CAD, coronary artery disease; cMDE, current major depressive episode; COMISA, comorbid insomnia and sleep apnea; n, number; PACs, premature atrial contractions; PHQ-9, patient health questionnaire, 9-items; PVCs, premature ventricular contractions; S.D., standard deviation; %, percentage.

The participant cohort was evenly divided into two groups, with 148 participants exhibiting suspected COMISA and 148 participants not exhibiting suspected COMISA. A total of 46 subjects received a cMDE diagnosis according to the MINI, indicating a prevalence of 15.54% (95% CI: 11.61%−20.18%). Within the group with suspected COMISA, 25 subjects were diagnosed with cMDE, resulting in a prevalence of 16.89% (95% CI: 11.24%−23.92%). In the group without suspected COMISA, 21 subjects were diagnosed with cMDE, yielding a prevalence of 14.19% (95% CI: 9.00%−20.87%). Table 1 provides a comprehensive summary of the analyzed subjects' characteristics, both overall and stratified by suspected COMISA status.

The prevalence of cMDE based on software, PHQ-9 cut-off of 10, and MINI interviews in the total sample and in the subgroups is presented in Table 2, and the accuracy of MEB-001 in detecting cMDE was assessed in the entire sample and OSA, suspected insomnia, and suspected COMISA, diagnoses groups is presented in Tables 3, 4. No statistically significant differences were observed between the subgroups in sensitivity (*p* = 0.686), specificity (*p* = 0.675), PPV (*p* = 0.691), or NPV (*p* = 1.000).

**Table 2:** Prevalence of cMDE based on different determination strategies.

**cMDE determination strategy**	**Total sample (*****n*** = **296)**		**Suspected COMISA (*****n*** = **148)**		**Not suspected COMISA (*****n*** = **148)**		**Unable to assess cMDE (*****n*** = **30)**	
	***N***	**%**	***N***	**%**	***N***	**%**	***N***	**%**
MEB-001	109	36.82%	51	34.46%	58	39.19%	-	-
PHQ-9 (cut-off ≥ 10)	120	40.54%	54	36.49%	66	44.59%	9	30.00%
MINI	46	15.54%	25	16.89%	21	14.19%	4	13.33%

**Table 3:** Screening performance of MEB-001 and PHQ-9 (cut- off ≥ 10) relative to MINI.

**Performancemeasurement**	**Total sample (*****n*** = **296)**					
	**MEB-001**			**PHQ-9** ≥**10**		
	**Point estimate**	**95% CI**		**Point estimate**	**95% CI**	
Sensitivity	84.78%	71.13%	93.66%	89.13%	76.43%	96.38%
Specificity	72.00%	65.99%	77.47%	68.40%	62.24%	74.11%
PPV	35.78%	26.83%	45.53%	34.17%	25.76%	43.38%
NPV	96.26%	92.44%	98.48%	97.16%	93.50%	99.07%
**Suspected COMISA (*****n*** = **148)**						
Sensitivity	88.00%	68.78%	97.45%	88%	68.78%	97.45%
Specificity	70.73%	61.85%	78.59%	64.23%	55.09%	72.67%
PPV	37.93%	25.51%	51.63%	33.33%	22.20%	46.01%
NPV	96.67%	92.44%	98.48%	96.34%	98.68%	99.24%
**Not suspected COMISA (*****n*** = **148)**						
Sensitivity	80.95%	58.09%	94.55%	90.48%	69.62%	98.83%
Specificity	73.23%	64.65%	80.69%	72.44%	63.81%	79.99%
PPV	33.33%	20.76%	47.92%	35.19%	22.68%	49.38%
NPV	95.88%	89.78%	98.87%	97.87%	92.52%	99.74%

CI, confidence interval; cMDE, current major depressive episode; COMISA, comorbid insomnia and sleep apnea; MINI, mini international neuropsychiatric interview; NPV, negative predictive value; n, number; PHQ-9, patient health questionnaire, 9-items; PPV, positive predictive value.

**Table 4:** Confusion matrices for MEB-001 and PHQ-9 (cut-off ≥ 10) in identifying current major depressive episode.

**Groups**		**MINI-based cMDE detected**	**MINI-based cMDE not detected**	**Total**
Full sample	MEB-001-based cMDE detected	39	70	109
	MEB-001-based cMDE not detected	7	180	187
	PHQ-9-based cMDE detected	41	79	120
	PHQ-9-based cMDE not detected	5	171	176
	Total	46	250	296
**Suspected COMISA**	MEB-001-based cMDE detected	22	32	54
	MEB-001-based cMDE not detected	3	94	97
	PHQ-9-based cMDE detected	22	32	54
	PHQ-9-based cMDE not detected	3	91	148
	Total	25	123	148
**Not Suspected COMISA**	MEB-001-based cMDE detected	17	41	58
	MEB-001-based cMDE not detected	4	86	90
	PHQ-9-based cMDE detected	21	45	66
	PHQ-9-based cMDE not detected	0	82	82
	Total	21	127	148

cMDE, current major depressive episode; COMISA, comorbid insomnia and sleep apnea; MEB-001, screening algorithm; MINI, mini international neuropsychiatric interview; PHQ-9, patient health questionnaire, 9 items (major depressive module).

The accuracy of the PHQ-9 (cut-off ≥10) was evaluated across the full sample and subgroups (Table 3). No statistically significant differences were observed between the subgroups in sensitivity (*p* = 1.000), specificity (*p* = 0.176), PPV (*p* = 0.849), or NPV (*p* = 0.665).

The paired screening performance of MEB-001 and PHQ-9 was directly compared using McNemar's test across the full sample and within COMISA subgroups.

In the full sample, no significant differences were observed between MEB-001 and PHQ-9 in the proportion of correctly identified cases of current major depressive episode (cMDE) for participants with cMDE (*p* = 0.414) or without cMDE (*p* = 0.198), as determined by the MINI.

Within the subgroup of participants with suspected COMISA, the proportion of correctly classified individuals also did not significantly differ for those with cMDE (*p* = 1.000) or without cMDE (*p* = 0.144).

Similarly, in the non-COMISA subgroup, no significant differences were found in the correct identification of participants with cMDE (*p* = 0.317) or without cMDE (*p* = 0.818).

Table 5 displays the agreement and disagreement rates between MEB-001 and PHQ-9, considering both the entire sample and each of the two COMISA subgroups.

**Table 5:** Agreement and disagreement rates between MEB-001 and PHQ-9 (cut-off ≥ 10) for detecting current major depressive episode.

**Groups**		**MINI-based cMDE detected**		**MINI-based cMDE not detected**	
		**PHQ-9-based cMDE detected**	**PHQ-9-based cMDE not detected**	**PHQ-9-based cMDE detected**	**PHQ-9-based cMDE not detected**
**Total Sample (N** **=** **296)**	MEB-001-based cMDE detected	33/71.74%	6/13.04%	179/71.60%	1/0.40%
	MEB-001-based cMDE not detected	2/4.35%	5/10.87%	26/10.40%	44/17.60%
**Suspected COMISA (N=148)**	MEB-001-based cMDE detected	18/72.00%	4/16.00%	87/70.73%	0/0.00%
	MEB-001-based cMDE not detected	0/0.00%	3/12.00%	11/8.94%	25/20.33%
**Not Suspected COMISA (N=148)**	MEB-001-based cMDE detected	15/71.43%	2/9.52%	91/78.45%	1/0.86%
	MEB-001-based cMDE not detected	2/9.52%	2/9.52%	15/12.93%	9/7.76%

cMDE, current major depressive episode; COMISA, comorbid insomnia and sleep apnea; MEB-001, screening algorithm; MINI, mini international neuropsychiatric interview; N, number; PHQ-9, patient health questionnaire, 9 items (major depressive module).

## 4 Discussion

This study evaluated the performance of a novel software, MEB-001, which combines physiological features with core depressive symptom reports and applies ML algorithms to detect cMDE in individuals with and without suspected COMISA referred for sleep studies at multiple U.S. clinics. Its screening accuracy was compared to the widely used PHQ-9, using a structured clinical diagnostic interview as the reference standard.

According to our results, a cMDE was diagnosed in 15.54% of the sample based on MINI interviews, with similar prevalence rates observed among participants with suspected COMISA (16.89%) and those without (14.19%). These rates are higher than those reported in general population studies, where point prevalence estimates for major depression typically range from 8 to 13% (Weinberger et al., 2018; Brody et al., 2018), indicating a heightened risk of depression within sleep clinic populations and underscoring the critical need for systematic screening in these settings. In contrast, screening tools indicated higher prevalence rates: the MEB-001 identified 36.82% of the total participants as having cMDE, with 34.46 and 39.19% in the respective subgroups. Similarly, using the PHQ-9 with a cut-off score of 10 or higher, 40.54% of participants screened positive for cMDE, including 36.49 and 44.59% in the two subgroups. It is well recognized that screening tools tend to overestimate the prevalence of conditions like cMDE compared to structured diagnostic interviews. This inflation occurs because screening instruments are designed to be highly sensitive to identify potential cases but may lack specificity, resulting in false positives. Consistent with this, both MEB-001 and PHQ-9 (with a cutoff ≥10) demonstrated high sensitivity (MEB-001: 84.8%; PHQ-9: 89.1%) but comparatively lower specificity (MEB-001: 72%; PHQ-9: 68.4%). Therefore, prevalence rates obtained through screening are generally higher than those derived from diagnostic assessments. This pattern aligns with previous research highlighting the tendency of screening measures to produce elevated prevalence estimates in epidemiological studies (Levis et al., 2019). Along with a higher sensitivity, a clinically effective screening instrument is characterized by a strong NPV, which indicates its ability to reliably rule out the condition when the test result is negative (Ben-Haim and Dacso, 2024; Trevethan, 2017). Both the MEB-001 and PHQ-9 demonstrated comparable NPVs (96.3 and 97.2% respectively) compared to the MINI-based diagnosis of cMDE, underscoring their utility in accurately identifying individuals without the disorder.

Consistent with our findings, a recent study demonstrated that in OSA patients, the coexistence of both insomnia symptoms and daytime sleepiness defines a distinct phenotype associated with a higher prevalence and greater severity of depressive symptoms. This work underscores the clinical heterogeneity of OSA and suggests that comorbid insomnia may amplify the risk of depression, highlighting the critical need for comprehensive symptom assessment to improve depression management in this population (Gabryelska et al., 2024a).

Recent evidence suggests a biological link between circadian clock dysregulation and depressive symptoms in individuals with OSA. In a study assessing circadian gene expression and depression severity using the Montgomery–Åsberg Depression Rating Scale (MADRS), a significant positive correlation was observed between morning expression levels of several circadian genes and the severity of depressive symptoms in OSA patients. Among these, morning expression of the PER1 gene emerged as an independent predictor of depression severity, even after adjusting for insomnia and chronotype-related variables. These findings support the hypothesis that disrupted circadian regulation may contribute to the pathophysiology of depression in OSA, offering a potential mechanistic explanation for the high comorbidity observed between these conditions (Gabryelska et al., 2024b).

Our findings herein demonstrate that both the MEB-001 and the PHQ-9 are robust screening instruments for cMDE in sleep clinic settings and exhibit comparable efficacy across diverse subgroups, encompassing both individuals with and those without suspected COMISA.

While both MEB-001 and PHQ-9 are valuable tools for screening cMDE, MEB-001 may outperform PHQ-9 in certain contexts due to its unique approach. Unlike the PHQ-9, which relies solely on self-reported symptoms, MEB-001 combines physiological features with core depressive symptom reports and applies ML algorithms for detection. This multi-dimensional approach helps mitigate limitations of self-report measures, such as underreporting due to stigma or social desirability bias. As a result, MEB-001 may offer more accurate detection in populations where mental health stigma is a barrier to honest disclosure. Additionally, by incorporating objective physiological data, MEB-001 can enhance screening accuracy in settings where literacy or familiarity with mental health concepts is limited, reducing misinterpretation common in traditional questionnaires. The use of objective, automated assessment may also help reduce legal and clinical risk—particularly related to responses to item 9 of the PHQ-9, which addresses suicidal ideation and may require urgent follow-up based on subjective interpretation. Furthermore, MEB-001 can lower the burden on clinicians by supporting efficient, standardized assessments. Therefore, MEB-001 offers distinct advantages in clinical and community contexts that demand sensitive, reliable, and scalable tools for the screening of depressive episodes.

It is noteworthy that the PPVs for both MEB-001 and PHQ-9 ≥10 were relatively low, at 35.8 and 34.2%, respectively. It is essential to acknowledge that the prevalence of a condition within the study population has a direct effect on the PPV. A diminished PPV suggests a higher incidence of false-positive results. When prevalence is low, even a highly specific test yields relatively few TP compared with FP, lowering PPV. In our sample (46 cMDE cases among 296 participants), this scarcity of true cases likely accounts for the modest PPV observed.

No significant differences were found in the screening performance of either the MEB-001 software or the PHQ-9 (cut-off ≥10) between subgroups (OSA, suspected insomnia diagnoses, and suspected COMISA). Both tools showed comparable sensitivity, specificity, PPV, and NPV across the groups. Additionally, direct comparisons of classification accuracy using McNemar's test revealed no significant differences between MEB-001 and PHQ-9 in the overall sample or within the subgroups. Our results showed that MEB-001 was juxtaposed with the PHQ-9 within subjects with or without suspected COMISA.

In literature, some studies have used ML to achieve the identification of subjects with depression using HR and HRV metrics and the input of the algorithm. For example, Shaw et al. used single-lead ECG recordings to differentiate individuals with comorbid OSA and depression from those with OSA without depression, achieving with this model an accuracy of 78.18%, sensitivity of 73.91%, specificity of 81.25%, and precision of 73.91% (Shaw et al., 2024). Another study analyzed 5-min HRV segments from sleep-stage ECG recordings in 40 MDD patients and 40 controls, reporting 85.9% sensitivity and 86.5% specificity using a Bayesian-optimized ensemble of extremely randomized trees (Geng et al., 2023). While these studies suggest the potential for identifying autonomic signatures of depression during sleep, they have notable limitations: both relied on the PHQ-9 for depression classification rather than structured diagnostic interviews, which are considered the gold standard; used cross-validation exclusively instead of independent test sets; and involved relatively small sample sizes.

Our study addresses these limitations by employing MINI-structured interviews as the reference standard, evaluating performance on an independent hold-out test set, and utilizing a significantly larger sample size, thereby enhancing the robustness and generalizability of our findings.

In addition to clinical utility, the potential cost-effectiveness of MEB-001 and its implications for resource allocation in sleep clinics warrant consideration. As an automated tool, MEB-001 could reduce the need for additional clinical staff time and follow-up appointments by streamlining depression screening within existing workflows, which is particularly valuable in resource-constrained settings. Its scalability may also support broader mental health integration into sleep medicine without imposing significant operational burdens. Looking ahead, future research should focus on the real-world deployment of MEB-001, including prospective validation in diverse clinical settings. Studies evaluating its long-term impact on patient outcomes, workflow efficiency, and healthcare resource utilization will be essential to fully establish its value and inform adoption in routine practice.

## 5 Limitations

Several methodological features of our study may help explain this discrepancy and the observed lack of a significant difference between groups. First, the diagnosis of COMISA in our study was based on a suspected rather than a confirmed clinical classification of insomnia. Insomnia symptoms were assessed via a brief self-reported questionnaire embedded in the intake form, without using validated instruments such as the ISI. Consequently, the insomnia component of COMISA may have been under- or misclassified, potentially introducing misclassification bias and diluting the observed differences in cMDE prevalence between groups. Future studies employing standardized and validated insomnia assessment tools could provide more accurate classification and strengthen the findings. Second, the broad inclusion criteria—encompassing a wide range of sleep disorders—may have contributed to a more heterogeneous sample, reducing the ability to detect contrasts specifically attributable to COMISA. Unlike studies that compare COMISA specifically to pure OSA or pure insomnia groups, our sample likely included individuals with overlapping or ambiguous sleep symptoms, further minimizing distinctions. Third, the use of MINI as the diagnostic reference for depression, while robust in validity, captures only categorical diagnoses of cMDE and may miss subthreshold depressive symptoms. Fourth, it is worth considering that both groups—COMISA and non-COMISA—may share common pathways of sleep fragmentation, circadian disruption, or fatigue that independently contribute to depressive risk, thereby flattening differences between them in this particular sample. Fifth, MEB-001 software was unable to provide a cMDE determination for roughly 9% of the subjects. This limitation could result in a small number of patients not receiving algorithm-based screening in a practical setting. Finally, although our analysis revealed no significant differences in performance between MEB-001 and PHQ-9, either in the full sample or within subgroups, this result may partly be attributable to the limited sample size and consequent reduction in statistical power.

## 6 Conclusion

Our results highlight the need for depression screening in the SCs. The failure of clinicians to identify acute depressive symptoms concurrent with sleep disturbances produces adverse clinical consequences, resulting in postponed diagnoses, management, and treatment of mental health disorders, notably MDD (Substance Abuse and Mental Health Services Administration, 2022). The absence of uniform mental health screening instruments in sleep clinics increases the probability of misdiagnosing and managing comorbid sleep disorders such as COMISA, which, due to the high prevalence of cMDE, can negatively impact treatment protocols and patient outcomes (Trevethan, 2017; Daccò et al., 2023).

Prior investigations and established guidelines advocate for the implementation of routine depression screening within sleep clinics to mitigate this concern. Consequently, certain authorities have underscored the necessity of standardized depression screening in these settings (Trevethan, 2017; Rosenberg, 2003). Conversely, prevailing guidelines stipulate psychological screening only for particular sleep-wake disorders, such as insomnia, rather than as a universally applied procedure (Trevethan, 2017; Rosenberg, 2003). However, a significant amount of evidence suggests that protracted periods of untreated depression and delayed administration of antidepressant therapy are correlated with suboptimal prognoses. Moreover, the inappropriate prescription of hypnotics and sedatives for the management of sleep disturbances in individuals with depression has been associated with an increased incidence of depression exacerbation and recurrence (Geng et al., 2023; Inada et al., 2021).

In light of the substantial prevalence of depressive, anxious, and stress-related symptomatology among individuals with COMISA (Smith et al., 2004; Lang et al., 2017), along with the complexities of its diagnosis, management, and treatment (Sweetman et al., 2019, 2017; Inada et al., 2021), a robust recommendation for depressive symptom screening is hereby asserted. The detection of these symptoms facilitates the capacity of clinicians to ascertain the optimal therapeutic regimen, thereby augmenting the efficacy of sleep and mental health management.

As previously discussed, traditional depression screening tools—such as self-report questionnaires—have played a valuable role in addressing the limitations of relying exclusively on clinical judgment, particularly when assessments are conducted by non-specialist providers. However, these tools also present well-documented limitations. They are vulnerable to subjective biases, such as the underreporting or minimization of symptoms, fluctuations in patient engagement or motivation, and the risk of incomplete or inaccurate responses. While generally less time-consuming than structured clinical interviews, self-report instruments still require clinicians to allocate time to explain the rationale for the questionnaire and provide clear instructions, which can pose a practical burden in high-demand clinical environments.

In light of these challenges, integrating automated depression screening into routine care has received growing support. Reliable tools that incorporate objective physiological signals offer a promising alternative to traditional self-report methods. By reducing subjective bias, improving diagnostic consistency, and easing clinician workload, such systems can enhance early detection and management of depression. Ultimately, embedding automated, evidence-based tools into clinical workflows may promote more efficient, equitable, and scalable mental health care. By reducing reliance on clinician-administered assessments, such tools can streamline screening processes and enable faster triage of at-risk individuals. The use of objective physiological data also promotes more consistent and standardized evaluations, minimizing subjectivity and helping to reach individuals who may underreport symptoms due to stigma or low mental health literacy. Furthermore, the scalability of automated systems supports broader implementation across diverse clinical settings, including those with limited access to mental health professionals. Together, these advantages position MEB-001 as a potentially valuable tool for enhancing depression detection and addressing mental health needs more effectively within routine medical care.

An important future direction involves the integration of MEB-001 into electronic health records and existing PSG systems. Embedding the tool directly into clinical workflows could enable prompt analysis of physiological and sleep data, with automated depression risk assessments delivered alongside standard PSG reports. Such integration would facilitate timely clinical decision-making, streamline documentation, and support coordinated care by making mental health screening results readily accessible to both sleep specialists and referring providers. Ultimately, interoperability with electronic health records and PSG platforms could enhance the scalability and impact of digital screening tools like MEB-001 in routine sleep medicine practice.

To conclude, the present analysis demonstrates that a novel software application, MEB-001—integrating both subjective and objective physiological data—offers an effective approach for screening cMDE in sleep clinic settings. The MEB-001 software showed comparable efficacy across different patient subgroups, including those with and without suspected COMISA, and performed on par with the PHQ-9, the current gold standard for depression screening in primary care and specialty settings. These findings support the potential utility of automated screening tools to facilitate the timely, efficient, and scalable detection of depression in sleep medicine populations, thereby addressing existing barriers to mental health assessment in these clinical environments. However, we recognize the critical importance of independent, external replication to validate the findings presented here. Future studies led by independent researchers are essential to confirm the robustness, generalizability, and clinical utility of MEB-001, ensuring unbiased evaluation and facilitating broader acceptance within the scientific and medical communities.

## Data Availability

The datasets presented in this article are not readily available because the dataset is Medibio Limited property. Requests to access the datasets should be directed to massimiliano.grassi@medibio.com.au.
